# IGF1R Signaling in Ewing Sarcoma Is Shaped by Clathrin-/Caveolin-Dependent Endocytosis

**DOI:** 10.1371/journal.pone.0019846

**Published:** 2011-05-17

**Authors:** Ana Sofia Martins, José Luis Ordóñez, Ana Teresa Amaral, Frans Prins, Giuseppe Floris, Maria Debiec-Rychter, Pancras C. W. Hogendoorn, Enrique de Alava

**Affiliations:** 1 Laboratory 20- Molecular Pathology of Sarcomas Laboratory, Centro de Investigación del Cáncer-IBMCC, Universidad de Salamanca-CSIC, Salamanca, Spain; 2 Department of Pathology, Leiden University Medical Center, Leiden, Netherlands; 3 Laboratory of Experimental Oncology, Catholic University of Leuven, Leuven, Belgium; 4 Department of Human Genetics, Catholic University of Leuven, Leuven, Belgium; The University of Kansas Medical Center, United States of America

## Abstract

Receptor endocytosis is critical for cell signaling. IGF1R mediates an autocrine loop that is de-regulated in Ewing Sarcoma (ES) cells. Here we study the impact of IGF1R internalization, mediated by clathrin and caveolin-1 (CAV1), in ES signaling. We used clathrin and CAV1-siRNA to interfere in clathrin- and caveolin-dependent endocytosis. Chlorpromazine (CPMZ) and methyl-beta-cyclo-dextrin (MCD) were also used in order to inhibit clathrin- and caveolin-dependent endocytosis, respectively. We analyzed IGF1R internalization and co-localization with clathrin and CAV1 upon ligand binding, as well as the status of the IGF1R pathway, cellular proliferation, and the apoptosis of interfered and inhibited ES cells. We performed a high-throughput tyrosine kinase phosphorylation assay to analyze the effects of combining the IGF1R tyrosine kinase inhibitor AEW541 (AEW) with CPMZ or MCD on the intracellular phospho-proteome. We observed that IGF1R is internalized upon ligand binding in ES cells and that this process is dependent on clathrin or CAV1. The blockage of receptor internalization inhibited AKT and MAPK phosphorylation, reducing the proliferative rate of ES cells and increasing the levels of apoptosis. Combination of AEW with CPMZ or MCD largely enhanced these effects. CAV1 and clathrin endocytosis controls IGF1R internalization and signaling and has a profound impact on ES IGF1R-promoted survival signaling. We propose the combination of tyrosine-kinase inhibitors with endocytosis inhibitors as a new therapeutic approach to achieve a stronger degree of receptor inhibition in this, or other neoplasms dependent on IGF1R signaling.

## Introduction

Receptor tyrosine kinases (RTKs) are involved in countless signaling pathways and their deregulation is involved in several diseases, in particular in cancer. Despite extensive study of these signaling cascades, until recently RTK internalization was believed to determine a down-regulation of RTK activity, ultimately leading to receptor degradation. However, increasing evidence indicates that endocytosis modulates and also sustains signaling transduction throughout the downstream targets of RTKs [1], [2], [3]. In most cell types, RTKs mainly internalize through clathrin-dependent internalization, converging in the formation of clathrin-coated membrane invaginations, or clathrin-coated pits (CCPs), in a series of highly regulated steps. Regarding caveolin-dependent internalization, receptor stimulation with caveolar budding leads to the formation of enclosed intracellular vesicles called “cavicles” [4]. Recently, it has been reported that the phosphorylation of CAV1 and dynamin-2 leads to caveolar fission [5]. Depending on the cellular context, internalized RTKs may then recycle back to the plasma membrane, recruit signaling proteins, thereby increasing active signaling from endosomes, or simply be degraded [1], [6], [7].

It has recently been reported that in lung cancer IGF1R endocytosis is triggered by ligand binding, causing IGF1R ubiquitination and internalization via clathrin-coated vesicles and/or caveolae [8]. Also, using an osteosarcoma model Sehat et al., have shown that IGF1R internalization is influenced by the ligand concentration [9], and Romanelli et al., have demonstrated that IGF1R internalization and recycling mediates the phosphorylation of AKT in glial progenitors [10]. Moreover, IGF1R is directly involved in the growth and survival of ES cells [11], [12], and given the relevance of RTK endocytosis in cell survival here we study the role of clathrin and/or CAV1 in ES IGF1R signaling.

## Results

### IGF1R is internalized by both clathrin- and CAV1-dependent mechanisms

In keeping with recent publications indicating that RTKs endocytosis regulates signaling transduction, we analyzed the role of IGF1R internalization in ES. This study was performed before and after ligand binding (IGF1) with the aim of exploring the two most common RTKs internalization mechanisms: clathrin- and caveolin-dependent endocytosis.

**Figure 1:**
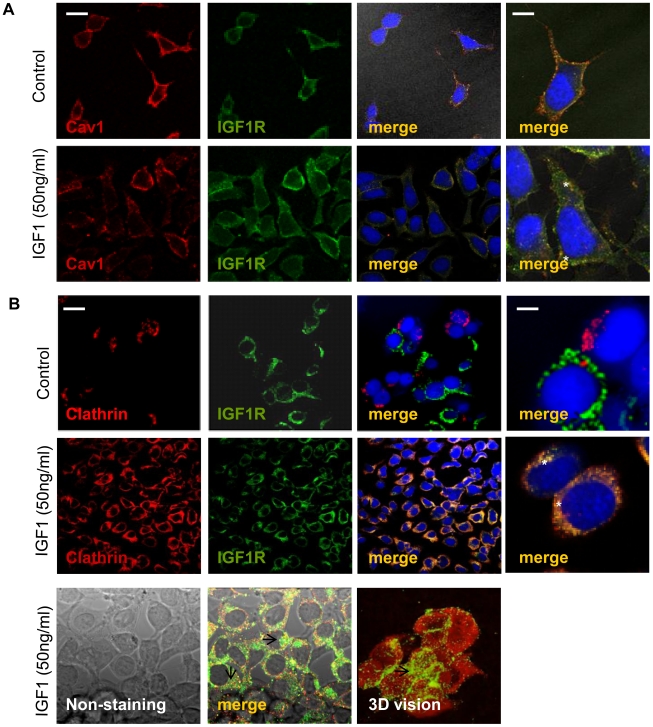
Confocal microscopy studies of IGF1R internalization in TC71 cells by Cav1-and clathrin-dependent mechanisms. Ewing's sarcoma TC71 cells were treated as described in the Material and Methods section. **A)** Cav1-dependent endocytosis studies. **B)** Clathrin-dependent endocytosis studies. Under basal conditions (non-stimulated with IGF1, see ‘control’ rows), IGF1R is confined to the membrane surface, while after IGF1 treatment it is internalized by clathrin- and caveolin1-dependent mechanisms, thus co-localizing with both clathrin/Cav1 inside the endocytic vesicles (see asterisks). The far right side panels show high magnifications for merge images. The bottom panels show additional images for IGF1R localization in the cytoplasm after IGF1-driven internalization. After ligand stimulation, IGF1R localizes both in ES cells membrane and cytoplasm, co-localizing with clathrin within the clathrin-coated pits (see arrows). The results obtained with CAV1 were similar to those shown for clathrin (data not shown). Scale bars represent 10–20 µm, left and right hand panels, respectively. Data presented is representative of 4–6 independent experiments.

We observed that under basal conditions (without IGF1 stimulation), IGF1R was present in the plasma membrane. However, upon ligand stimulation IGF1R was internalized by clathrin-dependent mechanisms (figure 1B) and by CAV1-dependent mechanisms, although to a lesser extent (figure 1A). Finally, we observed IGF1R co-localization with both clathrin and CAV1 in the endocytic vesicles (figure1 A and B).

**Figure 2:**
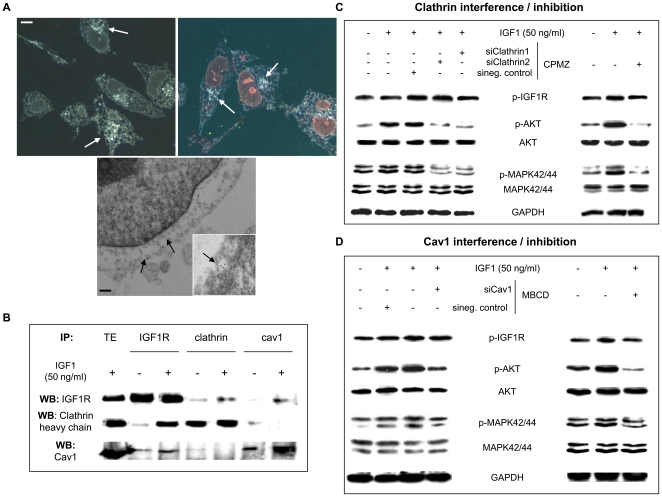
IGF1R internalization in TC71 cells by clathrin- and Cav1-dependent mechanisms and the effects of CAV1 or clathrin inhibition/interference in the IGF1R signaling pathway. **A**) Studies of RCM/EM microscopy show IGF1R in the ES cell cytoplasm (top panel, white arrows) and in the vesicles budding off the cell membrane (bottom picture, black arrows) after ligand binding. Scale bar represents 1 (RCM) or 0.1 µM (EM). **B**) IP/WB studies show that after stimulation with ligand (3rd, 5th and 7th lane), IGF1R co-precipitates with both clathrin (1^st^ row 5th lane; 2^nd^ row 3^rd^ lane) and with Cav1, although to a lesser extent (1^st^ row 7th lane, 3^rd^ row 3^rd^ lane). TE: total extract. **C**) WB detection of the IGF1R signaling pathway after CAV1 or D) clathrin interference/inhibition. Both approaches inhibited the IGF1R signaling pathway, inhibiting both AKT and MAPK42/44 phosphorylation. Data presented is representative of 3–5 independent experiments for the RCM/EM studies and 3–6 independent experiments for the IP/WB studies.

Moreover, since ES cells have scant cytoplasm, we were interested in confirming IGF1R signaling there, distinguishing this signaling from that performed by active IGF1R present in the cell membrane. As shown in figure 2, RCM/EM confirmed our previous results, clearly demonstrating that IGF1R was located in the cytoplasm (figure 2A top panel) and in vesicles budding off the cell membrane (figure 2A lower panel).

In order to confirm IGF1R internalization mediated by clathrin and CAV1, we performed co-IP studies. The results showed that after stimulation with IGF1, IGF1R co-precipitated with both clathrin and CAV1 (figure 2B). Again, co-precipitation occurred mainly with clathrin. Finally, we were able to confirm our initial hypothesis: in ES, IGF1R is indeed internalized by clathrin- and CAV1-dependent mechanisms.

### CAV1 and clathrin interference/inhibition leads to a blockade of IGF1R internalization

Here we tested the impact of clathrin or CAV1 interference and inhibition on IGF1R internalization and signaling using two different approaches: 1) specific siRNAs and 2) a drug approach, using MCD and CPMZ. MCD is known to deplete cholesterol, disrupting caveolae,[13] while CPMZ is able to inhibit clathrin-heavy-chain conformations.[14]

**Figure 3:**
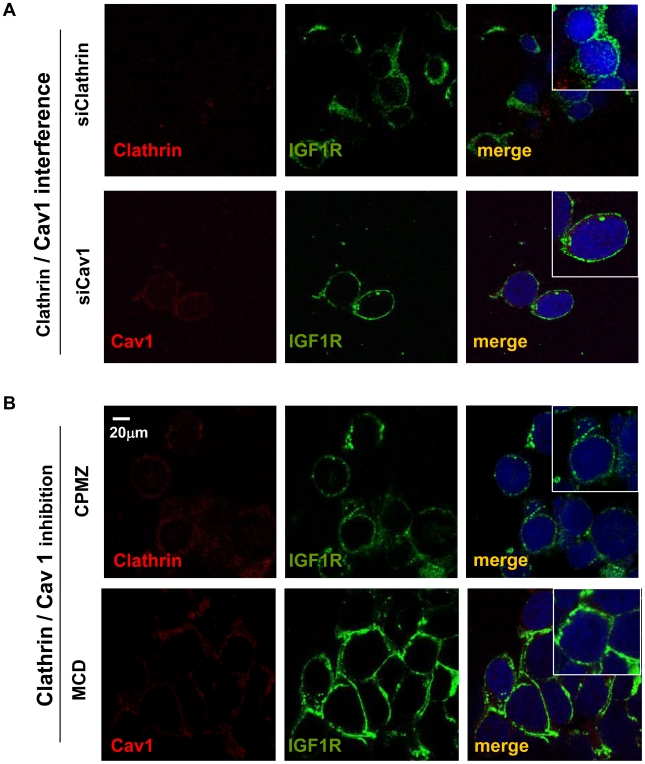
Confocal microscopy studies of IGF1R internalization after CAV1 or clathrin interference A)/inhibition B). Ewing's sarcoma TC71 cells were treated as described in the Material and Methods section. In comparison to control conditions (see figure 1), in the interfered/inhibited conditions IGF1R remains retained in the membrane after ligand stimulation. Scale bar represents 20 µm. Data presented is representative of 4–6 independent experiments.

Regarding clathrin and CAV1 interference, siRNAs effectively reduced CAV1 and clathrin protein levels (40–65% reduction) (see Figure S1 and figure 3A). Regarding clathrin and CAV1 inhibition through the use of MCD and CPMZ, we observed that the disruption of caveolae and the inhibiton of clathrin-heavy-chain conformations led to a decrease in IGF1R internalization (figure 3B). Overall, the interference or inhibition of endocytosis significantly decreased IGF1R levels of internalization, even after IGF1 stimulation, IGF1R being retained in the cell membrane. This shows that clathrin and CAV1 are key partners in IGF1R internalization.

### CAV1 and clathrin inhibition/interference impairs the IGF1R signaling pathway in ES cells

Several groups have shown that RTKs are frequently activated in endosomes, and so we were interested in determining the impact of the inhibition of clathrin and CAV1 endocytosis on IGF1R signalling. Accordingly, we also studied the consequent effects on cell growth and survival. In order to accomplish this, we compared the effects of CAV1 and clathrin inhibition and interference in 3 ES cell lines (TC71, A673 and A4573) with different levels of sensitivity to IGF1R inhibition, the A673 cell line being less sensitive, as reported previously by our group.[11]

**Figure 4:**
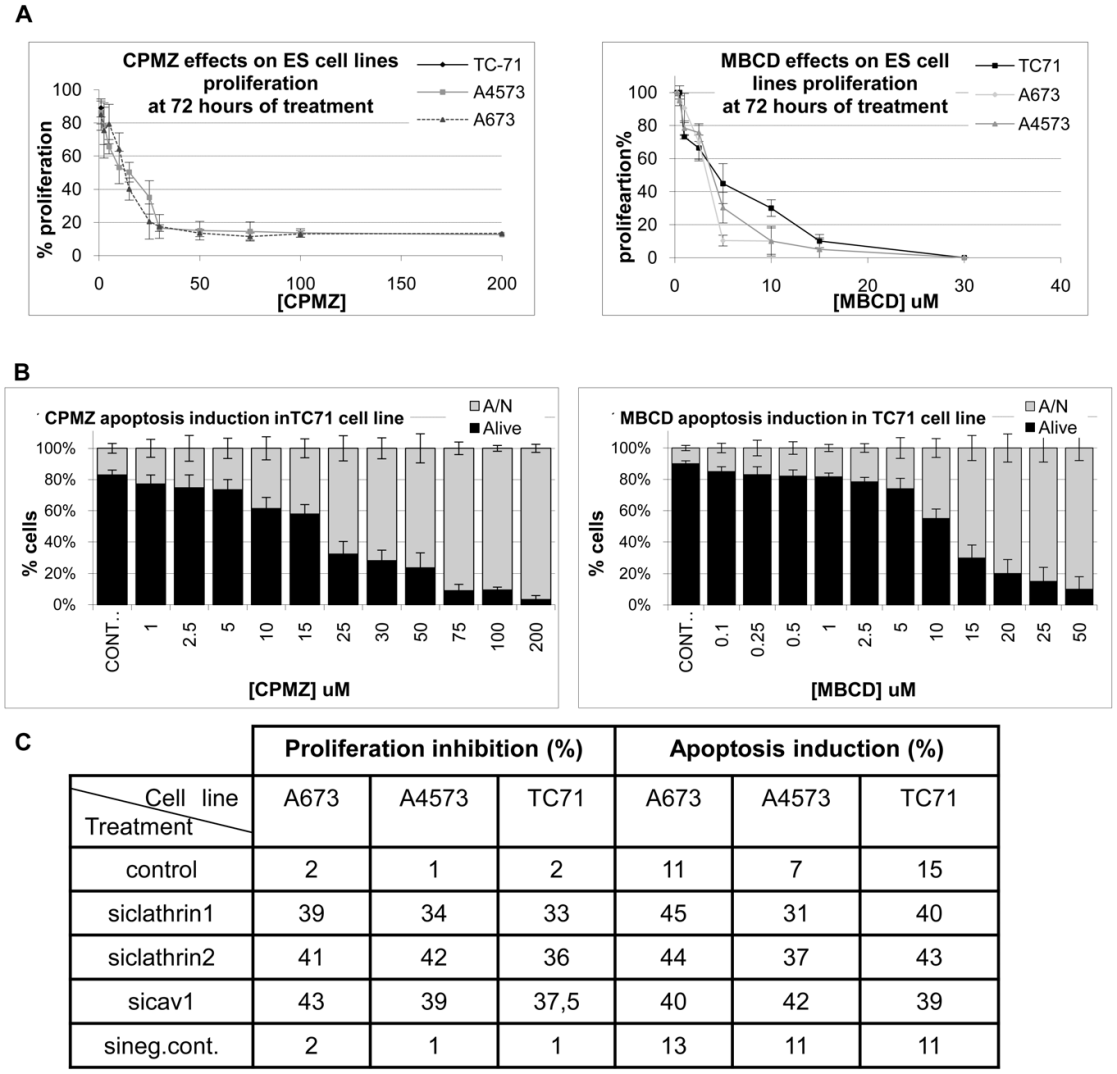
Effects of CAV1 or clathrin inhibition/interference in ES cell line proliferation and apoptosis. **A)** CAV1 or clathrin inhibition reduced ES cell line proliferation in a dose-dependent manner with an IC_50_ of proliferation similar in all ES cell lines tested (between 10–15 µM for CPMZ and 4–4.5 µM for MCD). **B)** The results obtained regarding the induction of apoptosis were similar to those obtained with the inhibition of proliferation in all cell lines (A673 and A4573 data shown in SD figure S3). A/N: Apoptotic and Necrotic cells. **C)** CAV1 or clathrin siRNAs induced around 40% of the inhibition of proliferation and 40–45% of apoptosis induction. Data presented as the average of 4–6 independent replicates.

Globally, we observed that inhibition with MCD and CPMZ was slightly more efficient than interference by siRNAs in all 3 cell lines tested. Nevertheless, both approaches led to the inhibition of IGF1R signaling, with a reduction in AKT and MAPK phosphorylation of more than 60% (p<0.05) (figures 2C and D and Figure S2 and Figure S3). No changes regarding IGF1R phosphorylation were detected. The reduction in MAPK was less evident in the case of CAV1 inhibition. Regarding CAV1 or clathrin inhibition (treatment with MCD and CPMZ), we detected a clear reduction in proliferation, with a dramatic induction of apoptosis in a dose-dependent manner. The IC_50_ of proliferation was similar in all cell lines (between 10–15 µM for CPMZ and 4–4.5 µM for MCD) (figure 4A). Similar results were obtained with the induction of apoptosis (figure 4B, and Figure S4). Additionally, another ES cell line quite independent of IGF1 showed higher IC_50_ proliferation values, showing that CPMZ and MCD toxicity is actually related to IGF1R endocytosis inhibition and not due to non-specific secondary effects of these drugs (Figure S5). Regarding the siRNA approach, we obtained around 40% of proliferation inhibition and 40–45% of apoptosis induction (figure 4C) for CAV1/clathrin interference at 72 hours of treatment (figure S4 for A673 and A4573 apoptosis data). Interestingly, both treatments mainly induced late apoptosis, few cells undergoing necrosis.

### CAV1 or clathrin inhibition improves drug-induced IGF1R tyrosine kinase inhibition

**Figure 5:**
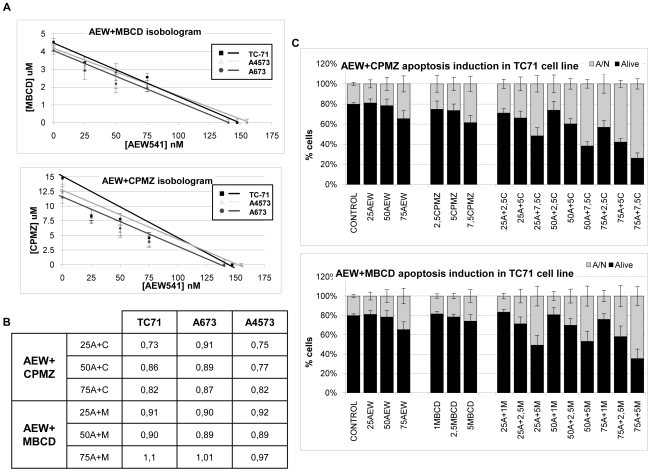
Effects of IGF1R tyrosine kinase inhibition combined with CAV1 or clathrin inhibition on ES cell proliferation and apoptosis. **A**) Isobolograms of AEW combined with MCD/CPMZ. **B**) Combination index (CI) of AEW combined with MCD/CPMZ. AEW combined with MCD shows additive effects in the inhibition of ES cell line proliferation (CI around 1). In contrast, regarding AEW combined with CPMZ the effects were synergistic (CI between 0.73–0.86) in the A4573 and TC71 cell lines, and only additive in the A673 cell line. **C**) AEW combined with MCD/CPMZ had additive effects on the induction of apoptosis in ES cell lines, mainly inducing late apoptosis, with few cells undergoing necrosis. A: AEW, C: CPMZ and M: MCD. Data presented as the average of 4–6 independent replicates.

Previous observations obtained in our own work indicated that IGF1R signaling can be enchanced after clathrin- and/or caveolin-dependent internalization. Accordingly we were interested in testing a new drug combination that has never been assayed before in ES. Here we tested the effect of the combination of the commonly used tyrosine kinase inhibitor, AEW541(AEW), with the previously evaluated endocytosis inhibitors CPMZ and MCD on the levels of proliferation (figure 5A and B) and the induction of apoptosis (figure 5C).

**Figure 6:**
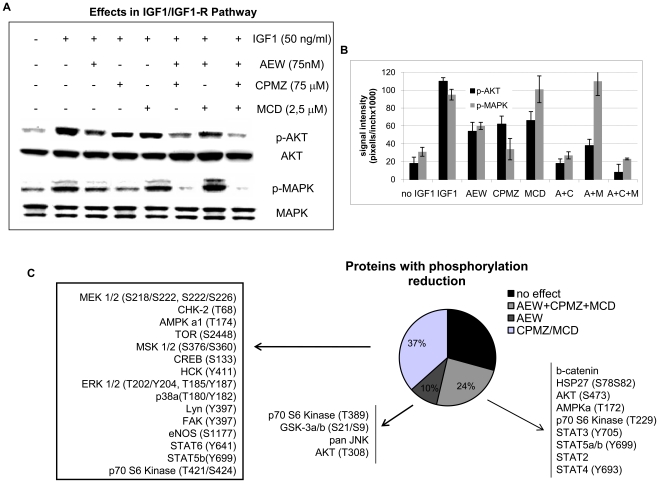
Effects of IGF1R tyrosine kinase inhibition combined with CAV1 and/or clathrin inhibition on the ES cell phospho-proteome. **A**) AKT and MAPK42/44 protein levels and **B**) densitometric evaluation. The reduction in the phosphorylation of AKT and MAPK42/44 was over 60%. **C**) Graphic representation of the most relevant changes in protein phosphorylation. Some of them underwent the strongest reduction in phosphorylation simply with CPMZ/MCD treatment (p38, ERK1/2, MEK1/2, or TOR). However, others needed the triple combination in order for the lowest levels of activation to be obtained (such as AKT, HSP27, or AMPKa1). A: AEW, C: CPMZ and M: MCD treatments; + control: IGF1 stimulation without drug treatment; - control: basal conditions, without IGF1 stimulation. Data presented is representative of 3–5 independent experiments for the WB studies and 3 independent experiments for the phospho kinase array.

As illustrated in the isobolograms shown in figure 5A, AEW541 in combination with MCD exerted additive effects on the cell lines tested (figure 5A). Regarding the combination of AEW with CPMZ, the effects were synergistic in two ES cell lines (A4573 and TC71) (with CI between 0.73–0.86, as shown in the table of figure 5B), and additive in the case of the A673 cell line, which is resistant to IGF1R inhibition and consequently less dependent on the IGF1R signaling pathway, as previously shown by our group [11], [12]. As expected, we observed a marked reduction in AKT and ERK1/2(MAPK) phosphorylation (figure 6A y B and Figure S6), in accordance with our data on the effects of CPMZ/MCD on IGF1R signaling (figure 2C and D). AKT phosphorylation elicited a reduction of around 50% when treated with AEW, and 35–40% when treated with CPMZ/MCD. The triple combination almost completely abolished AKT phosphorylation. Regarding MAPK, its phosphorylation was only reduced with AEW or CPMZ treatment (45 and 65% reduction, respectively), undergoing a higher reduction with the AEW+CPMZ combination. This suggests that CAV1 internalization probably does not mediate MAPK signaling.

In this study we detected changes in the phosphorylation of several other proteins (figure 6C), such as p38, TOR, MEK1/2, among others. Interestingly, 37% of all proteins underwent the strongest reduction in phosphorylation with CPMZ or MCD treatment and only 10% did so with AEW, while 24% underwent the highest reduction in phosphorylation with the triple treatment. These results suggest that AEW alone is able to inhibit IGF1R signaling but that this process may be enhanced by the combination with endocytosis inhibitors such as CPMZ and/or MCD.

## Discussion

It has been described that IGF1R upregulation promotes cell survival and proliferation in many types of cancer, including carcinomas such as prostate [15], breast [16] and lung carcinomas [17], [18], and also in sarcomas [11], [19]. Moreover, IGF1R plays an important role in the transformation, motility and metastasis of cancer cells [20], [21], [22], thus offering an attractive target in cancer therapy [22], [23]. Currently, there are 12 anti-IGF1R strategies undergoing clinical evaluation, including monoclonal antibodies and small-molecule tyrosine kinase inhibitors [22], [24]. Nonetheless, we believe that the full mechanism of IGF1R signalling remains to be clarified.

IGF1R is able to activate several signalling pathways through the phosphorylation of different intracellular proteins, including insulin receptor substrate-1 (IRS-1) and the Shc proteins [25], [26], [27], among many others. Several studies have previously shown that following hormone binding, IGF1R is internalized through endocytosis [28], [29], culminating in the formation of early endosomes containing the internalized active receptor [30], [31]. Also, Baserga et al. [32], demonstrated a strict correlation between the protein levels of CAV1 and IRS-1 in mouse embryo fibroblasts (MEFs) following deletion of the CAV1 gene (KO cells), where they found that IRS-1 was also downregulated. Recently, Sehat et al., confirmed this hypothesis in an osteosarcoma model [9], demonstrating that IGF1R undergoes ligand-dependent internalization through both clathrin-coated vesicles and caveolae. Thus, in this model the ligand concentration determines the trafficking pathway (caveolin-dependent or clathrin-dependent).

In line with this, we decided to study the involvement of IGF1R internalization in ES signaling and its possible targeting benefits. Initially, we observed that IGF1R was being internalized in ES cell lines after ligand binding, mainly due to clathrin–dependent mechanisms. We were able to show that i) clathrin or CAV1 inhibition/interference blocks IGF1R internalization, ii) IGF1R signaling in ES largely depends on receptor internalization, and iii) the pro-survival and anti-apoptotic effects of IGF1R signaling are considerably reduced when internalization is blocked.

**Figure 7:**
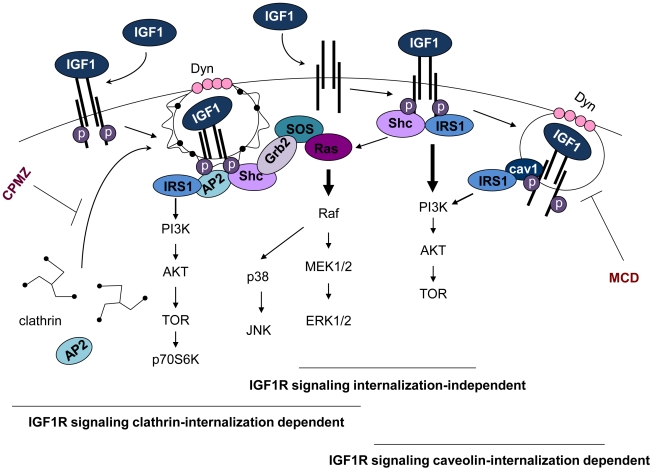
Simplified model for IGF1R signaling in ES. Globally, after ligand binding IGF1R can signal directly from the membrane (IGF1R signaling internalization-independent), with a major degree of IRS1/PI3K/AKT signaling activation and a smaller enhancement of the Ras/Raf/MEK/ERK signaling pathway, or, when internalized either by clathrin and/or caveolin-dependent mechanisms, it can signal from vesicles. In the case of clathrin-dependent endocytosis, IGF1R is recruited to CCPs through interaction of its cytoplasmic tails with the protein adaptor AP2, which in turn recruits clathrin to the plasma membrane and promotes its assembly into a lattice network. Through protein-protein interaction, clathrin and AP2 also bind several proteins of the endocytic machinery, such as dynamin, which regulates the fission of the CCPs with the consequent formation of CCVs, and GRB2, which recruits and binds the SOS protein and the IGF1R-phosphorylated protein Shc, activating the RAS/RAF/MEK/ERK and p38MAPK signaling pathways. To a lesser extent, it also activates the IRS1/PI3K/AKT signaling pathway. In the case of caveolin-dependent endocytosis, IGF1R is recruited to caveolae, promoting caveolin phosphorylation and the subsequent recruitment of dynamin, which promotes caveolar budding and the formation of cavicles. Cav-1 phosphorylation phosphorylates IRS-1, enhancing the activation of thePI3K/AKT signaling pathway. The thickness of the arrows indicates the prevalence of signaling pathway activation. CPMZ treatment inhibits the formation of CCP by inhibiting clathrin-heavy-chain conformations and MCB inhibits the formation of cavicles by depleting cholesterol, disrupting the caveolae.

The results obtained support the initial hypothesis to the effect that IGF1R signaling is enhanced after receptor internalization in ES. This mechanism allows access of upstream active kinases to direct downstream targets, thus permitting higher signaling specificity through the different subcellular compartments. Based on our results, we propose that in ES, after ligand binding, IGF1R is internalized under two different mechanisms: i) one dependent on clathrin and ii) the other caveolin-dependent (figure 7). However, it was predictable that IGF1R would be internalized by clathrin-dependent mechanisms, since this mechanism had already been reported for several RTKs, including IGF1R, in other tumor types [29], [30], [31]. The observation of IGF1R internalization through caveolin-dependent mechanisms clearly confirms previously published data [9] and provides new insight into the regulation of RTKs throughout internalization in ES. Moreover, Notario's group has recently shown that CAV1 is a target of EWS/FLI1 and that it is determinant for the oncogenic phenotype and tumorigenicity of ES cells. In fact, this particular study [33] shows that CAV1 knockdown reduces the growth of ES cell-derived tumors in nude mice xenografts. Also in that study the reactivation of CAV1 expression in ES cells enabled the rescue of the original oncogenic phenotype. Furthermore, Toretsky's data [34] showed that IGF1R is required for the EWS/FLI-1 transformation of fibroblasts.

Taken together, all these findings lead to the hypothesis that these molecular hits could be promoting ES tumorigenicity. Our results suggest that IGF1R internalization in ES is under a highly controlled spatial and temporal regulation, combining both clathrin and CAV1 mechanisms and underscoring the role of CAV1 in RTK endocytosis.

In keeping with the observations discussed previously, together with Podar's studies in multiple myeloma [35] and our results from the phospho-array assay, we suggest that IGF1R clathrin-endocytosis mainly regulates the RAS/RAF/MEK/ERK pathway in ES. Moreover, we believe that endocytosis further improves the IRS1/PI3K/AKT signaling pathway, since CPMZ treatment also reduced AKT, TOR and p70S6K phosphorylation. Nonetheless, we assume that the PI3K/AKT pathway undergoes its strongest activation either directly from the membrane, with IRS1 activation independent of IGF1R internalization (like the one reported by Chow et al.,[36]) or with an extra regulation, through caveolin-dependent internalization [13]. The data supporting this assumption show that the phosphorylation of AKT is already reduced by about 50% with AEW treatment (confirming that IGF1R signaling pathway activation is not totally dependent upon internalization), even though it showed a large improvement with the triple treatment, 80% of inhibition being reached. More importantly, the residues whose phosphorylation is reduced in the AKT, TOR, AMPK and p70S6K proteins are different, depending on the treatment used (CPMZ vs MCD vs combination). This constitutes additional evidence that endocytosis not only provides additional control over the “quantity” of RTKs signaling, but also over the “quality and selectivity” of such signaling. The model suggested here (see figure 7) provides new insight into the selectivity and specificity of IGF1R signaling driven from different cellular compartments.

With the studies on the effects of AEW541 and the CPMZ/MCD combination on ES cells, here we demonstrate that IGF1R tyrosine kinase inhibition is improved when combined with CAV1 or clathrin inhibition, reducing the levels of proliferation and the induction of apoptosis in ES cell lines. The effects of the drug combination had an impact on the phosphorylation status of several proteins, namely AKT, SRC p38, TOR, MSK1/2, MEK1/2, among others, confirming once again that IGF1R signaling depends on its internalization. Also, proteins such as HSP27, AKT, β-catenin, AMPKa and STAT2/4/3 exhibited the highest reduction in phosphorylation with the triple treatment, supporting the idea of combining TK inhibitors with internalization blockers in order to decrease signaling. It should be noted that RTK-blocking antibodies would, in theory, only block receptors located in the cellular membrane, not affecting the receptors already internalized. In this case, the small-molecule tyrosine kinase inhibitors would be a better choice since they would diffuse to the cytoplasm and also inhibit some of the receptors within the endocytic vesicles.

In conclusion, here we show that blockade of IGF1R internalization, either by clathrin or caveolin1, inhibits IGF1R signalling in ES cells. Accordingly, we provide new evidence regarding the role of cav1/clathrin endocytosis in IGF1R internalization. Finally, we consider the combination of TK inhibitors with endocytosis inhibitors as a major and plausible strategy to reduce ES cell proliferation through IGF1R signaling.

The results shown here are focused on Ewing Sarcoma, but it is extremely likely that they may find a more general application in sarcomas, and in several subsets of other more prevalent neoplasms that depend largely upon IGF1R survival signals (i.e. subsets of lung or breast carcinoma), therefore increasing the potential impact of our findings for sarcoma therapy.

## Materials and Methods

### Cell lines

The A673, TC-71, SK-ES-1, A4573 and TTC466 cell lines were obtained and maintained as previously described [11], [12], [37]. All cells were free of *Mycoplasma*, as screened with the MycoAlert Mycoplasma Detection kit (Lonza, Rockland, ME).

### Drugs

AEW541 (AEW) was kindly provided by Novartis Pharma AG, Basel, Switzerland. Chlorpromazine (CPMZ) and Methyl-β-cyclodextrin (MCD) were purchased from Sigma-Aldrich (St Louis, MO).

### siRNA

Caveolin1 pre-designed siRNA, clathrin-validated siRNAs and Silencer negative control siRNA were purchased from Ambion (Austin, TX). Cell lines were treated with 100 nM siRNAs, as previously described,[12] using lipofectamine 2000 (Invitrogen, Carlsbad, CA), according to the manufacturer's instructions.

### Immunofluorescence (IF) and Confocal Microscopy (CM)

Cells were grown overnight on sterile glass slides (covered with gelatine 0.1%), with serum-free medium and then stimulated with IGF1 for 15 minutes. The glass slides were washed with PBS and fixed with 2%(w/v) formaldehyde (Sigma-Aldrich) for 30 min. Cells were permeabilized in PBS/Triton x-100 0,1% (Invitrogen) for 30 minutes, incubated with PBS 50 nM NH_4_CL for 10 minutes, blocked for 30 minutes with PBS 0.2% BSA (Sigma Aldrich), and incubated overnight at 4°C with one of the following primary antibodies; rabbit polyclonal antibody against caveolin1 (Abcam), or rabbit polyclonal antibody against clathrin (Abcam), or mouse monoclonal antibody against clathrin (Affinity Bioreagents). After three washes in PBS, the slides were incubated with one of the following secondary antibodies: chicken anti-rabbit AlexaFluor594 (Invitrogen) or goat anti-rabbit Cy3 (Jackson ImmunoResearch Laboratories, Inc, West Grove, PA) or goat anti-mouse AlexaFluor594 (Invitrogen), for 45 min, and washed three times. Then, all the slides were incubated with rabbit polyclonal antibody against IGF1R (cell signalling) for 1 hour, washed three times, and incubated with chicken anti-rabbit Alexa 488 (Invitrogen) for 1 hour. Following this, cells were washed three times and nuclear DNA was counterstained with DAPI (2.5 mg/mL) (Invitrogen). Cells were mounted using the Slowfade Gold Antifade reagent (Invitrogen) or Vectashield H-1000 medium (Vector). A Zeiss LSM510 confocal microscope or a Leica inverted fluorescence microscope connected to a digital video camera (Leica DC100) were used to obtain confocal or fluorescence images. Confocal image analysis was performed using the LSM 5 Image Browser (program version 2.8). 4–6 independent experiments were performed and the final data presented selected as the most representative of the results obtained.

### Immuno-Electron (EM) and Reflection Contrast Microscopy (RCM)

After treatment with IGF1, cells were fixed for 15 minutes in 2% paraformaldehyde/0.1% glutaraldehyde and spun down at 7500 rpm for 5 min. Cells were embedded in 12% gelatine, spun down and fixed over-night at 4°C (with a 2.3 M sucrose solution on top of them). Cell pellets were cut on an ultramicrotome (Leica, Germany) at 70 nm thickness and sections were placed on carbon-coated hexagonal grids. The sections were incubated with rabbit polyclonal antibody against IGF1R overnight at 4°C, washed with PBS, incubated with ProteinA-Gold-10 nm antibody for 2 h at room temperature, and embedded in methyl-cellulose-0.3% uranyl acetate for 5 min. Photos were taken with a JEOL JEM1011 Transmission Electron Microscope connected to a digital video camera (MegaView III) or a Leica DM/RB microscope with an RCM module and a DFC-420C digital camera. 3–5 independent experiments were performed and the final data presented selected as the most representative of the results obtained.

### Western Blotting (WB) and Immunoprecipitation (IP)

IP and WB studies were performed as previously reported [11], [12]. The antibodies used were: anti-AKT, anti-p-AKT (Ser473), anti-MAPK42/44, anti-p-MAPK42/44 (Thr202/Tyr204) (all from Cell Signaling, Danvers CA), anti-caveolin1 (Abcam), anti-clathrin (Affinity Bioreagents) and anti-GAPDH (Abcam). 3–6 independent experiments were performed and the final data presented selected as the most representative of the results obtained.

### Proliferation studies

The dose-response proliferation levels of the cell lines subjected to drug treatment were analyzed to determine the IC_50_ of proliferation (control treatment performed using the vehicle for CPMZ and MCD). The percentage of proliferation inhibition induced by the specific CAV1/clathrin siRNAs was also evaluated. 4–6 independent experiments were performed and the final data are presented as averages of all replicates. To determine the proliferation rate, we used the MTT method, as previously described.[11]

### Apoptosis

The apoptosis and necrosis indices were assessed by flow cytometry, as previously described.[11] 3–5 independent experiments were performed, the final data being presented as averages of all replicates. Briefly, cell pellets were washed twice with PBS and stained with Anexin V for 15 minutes, washed, and then stained with Propidium Iodide (IP) for 5 minutes. Cells were acquired in a FACS Calibur cytometer. Cells with doble staining were considered as Apoptotic and Necrotic (A/N) while cells with no staining were considered Alive. 4–6 independent experiments were performed and the final data are presented as averages of all replicates.

### Isobolographic Analysis

The effects of the combination of AEW with CPMZ or MCD were analyzed using Loewe's isobolographic analysis, revised by Steel and Peckman, as previously described [11]. Briefly, for isoeffective doses of a two-drug combination (dA + dB) and the individual drugs alone (DA and DB), combinations having a combination index (CI)>1 were considered antagonistic; those with a combination index = 1 were additive, and those with a combination index <1 were synergistic, the combination index being  =  dA/DA + dB/DB. The CI values for each condition were calculated using the IC_50_ of proliferation as the isoeffective point (using Origin 6.0 to plot the data). Isobolograms were acquired by plotting the IC_50_ of AEW on the Y-axis and the IC_50_ of CPMZ or MCD on the X-axis.

### Phospho-kinase array

The effects of the AEW with CPMZ and/or MCD combinations were analyzed at the level of global kinase phosphorylation, using the dot-blot Proteome Profiler™ Array Human Phospho-Kinase Array Kit (R&D Systems), according to manufacturer's instructions. Briefly, cells were treated with 75 nM AEW, 7.5 mM CPMZ and/or 2.5 mM MCD for 1 hour, stimulated with IGF1 for 15 minutes, and lysates were prepared with the appropriate lysis buffer (PBS 1x, 1 mM Na_2_EDTA, 1 mM EGTA, 50 mM NaF, 1%Triton x-100, 5 mM Na_3_VO_4_ and protease inhibitor cocktail tablet (Roche). 400 µg of protein was used. The signals from the membranes were detected as described for WB and the levels of chemiluminescence of each spot were evaluated by 2D-densitometry using the AIDA^©^. software (Fujifilm-Raytest, Straubenhardt, Germany). 3 independent experiments were performed and the final data are presented as averages of all replicates.

### Statistical Analyses

One-Way ANOVA for independent samples was performed, using the VassarStats web site for Statistical Computation.

## Supporting Information

Figure S1 **siRNA knockdown efficiency studies.** Ewing's sarcoma cells were grown until they reached 30–40% confluence, treated with CAV1 or clathrin siRNAs, and then proteins were extracted and WB performed, as described in the Materials and Methods section.** A**) WB detection of clathrin and Cav1 protein levels. **B**) Densitometric analysis of the WBs. The siRNAs used effectively reduced CAV1/clathrin protein levels, with a 40–65% reduction, depending on the ES cell line treated. UTF: untransfected; mock: transfected without siRNA. 3 independent experiments were performed and the final data presented selected as the most representative of the results obtained -panel A and as averages of all replicates-panel B.
(TIF)

Figure S2 **Densitometric analyses of WB shown in** **figure 2C and D** **of effects of CAV1 or clathrin inhibition/interference on the IGF1R signaling pathway.** The inhibition of phosphorylation reached levels of over 60% reduction (p<0.05). The results obtained with the A673 and A4573 cell lines were identical to those described for TC71 (data not shown). Data presented is representative of 3–6 independent experiments.
(TIF)

Figure S3 **Effects of CAV1 or clathrin inhibition/interference on the IGF1R signaling pathway.** Ewing's sarcoma TC71 cells were starved overnight, stimulated with IGF1 (50 ng/ml) for 15 minutes, and then proteins were extracted and WB was performed, as described in the Material and Methods section. **A)** Effects of clathrin inhibition/interference. **B**) Effects of Cav1 inhibition/interference. The phosphorylation of AKT and MAPK proteins in siRNA- or drug-treated cells without IGF1 treatment showed that siRNA or drug treatment alone do not interfere with protein phosphorylation. 3 independent experiments were performed and the final data presented selected as the most representative of the results obtained.
(TIF)

Figure S4 **Effects of CAV1 or clathrin inhibition on apoptosis in the ES cell lines A673 and A4573.** Ewing's sarcoma cells were grown until they reached 30–40% confluence, treated with MCD/CPMZ for 72 hours, and then apoptosis was measured by flow cytometry, as described in the Materials and Methods section. Drug treatment induced apoptosis in a dose-dependent manner in all ES cell lines, mainly inducing late apoptosis, with few cells undergoing necrosis. Data presented as the average of 4–6 independent replicates.
(TIF)

Figure S5 **Effects of CAV1 or clathrin inhibition on an IGF-1-independent ES cell line.** Ewing's sarcoma TTC466 cells were grown until they reached 30–40% confluence, treated with MCD/CPMZ for 72 hours, and then proliferation was measured with the MTT assay, as described in the Materials and Methods section. **A)** Characterization of the IGF1 proliferation-driven signaling dependence of the TTC466 cell line, as already published by Martins el al. Cancer Res. 2008; 68(15):6260–70. **B)** CPMZ or MCD treatment. CAV1 or clathrin inhibition hardly affected ES cell line proliferation. The IC_50_ of proliferation was much higher than with the 3 other cell lines studied (5–10x), showing that the toxicity of CPMZ and MCD is related to IGF1R endocytosis inhibition and not due to non-specific secondary effects of these drugs. Data presented as the average of 4-6 independent replicates.
(TIF)

Figure S6 **Effects of IGF1R tyrosine kinase inhibition combined with CAV1 and/or clathrin inhibition on the ES cell phospho-proteome.** Images of the p-array membranes of all samples studied. A: AEW, C: CPMZ and M: MCD treatments; + control: IGF1 stimulation without drug treatment; - control: basal conditions, without IGF1 stimulation. Data presented is representative of 3 independent experiments.
(TIF)
