# Supplementary figures and images for: IGF1R Signaling in Ewing Sarcoma Is Shaped by Clathrin-/Caveolin-Dependent Endocytosis
Source: PLoS One. 2011 May 17;6(5):e19846. doi: 10.1371/journal.pone.0019846 (PMC3096649; doi:10.1371/journal.pone.0019846)

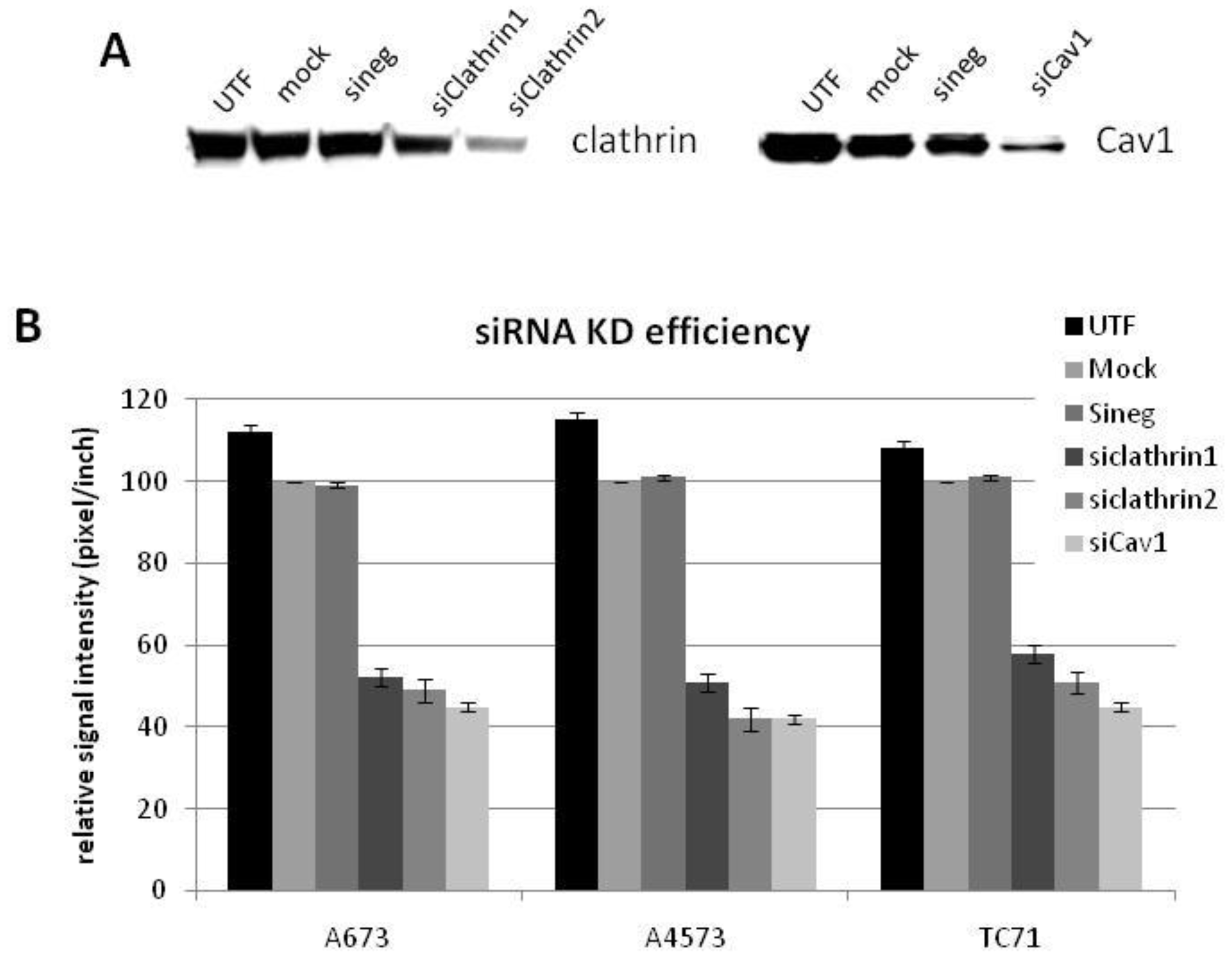

Supplement: Figure S1 — siRNA knockdown efficiency studies. Ewing's sarcoma cells were grown until they reached 30–40% confluence, treated with CAV1 or clathrin siRNAs, and then proteins were extracted and WB performed, as described in the Materials and Methods section. A) WB detection of clathrin and Cav1 protein levels. B) Densitometric analysis of the WBs. The siRNAs used effectively reduced CAV1/clathrin protein levels, with a 40–65% reduction, depending on the ES cell line treated. UTF: untransfected; mock: transfected without siRNA. 3 independent experiments were performed and the final data presented selected as the most representative of the results obtained -panel A and as averages of all replicates-panel B. (TIF) [file pone.0019846.s001.tif]

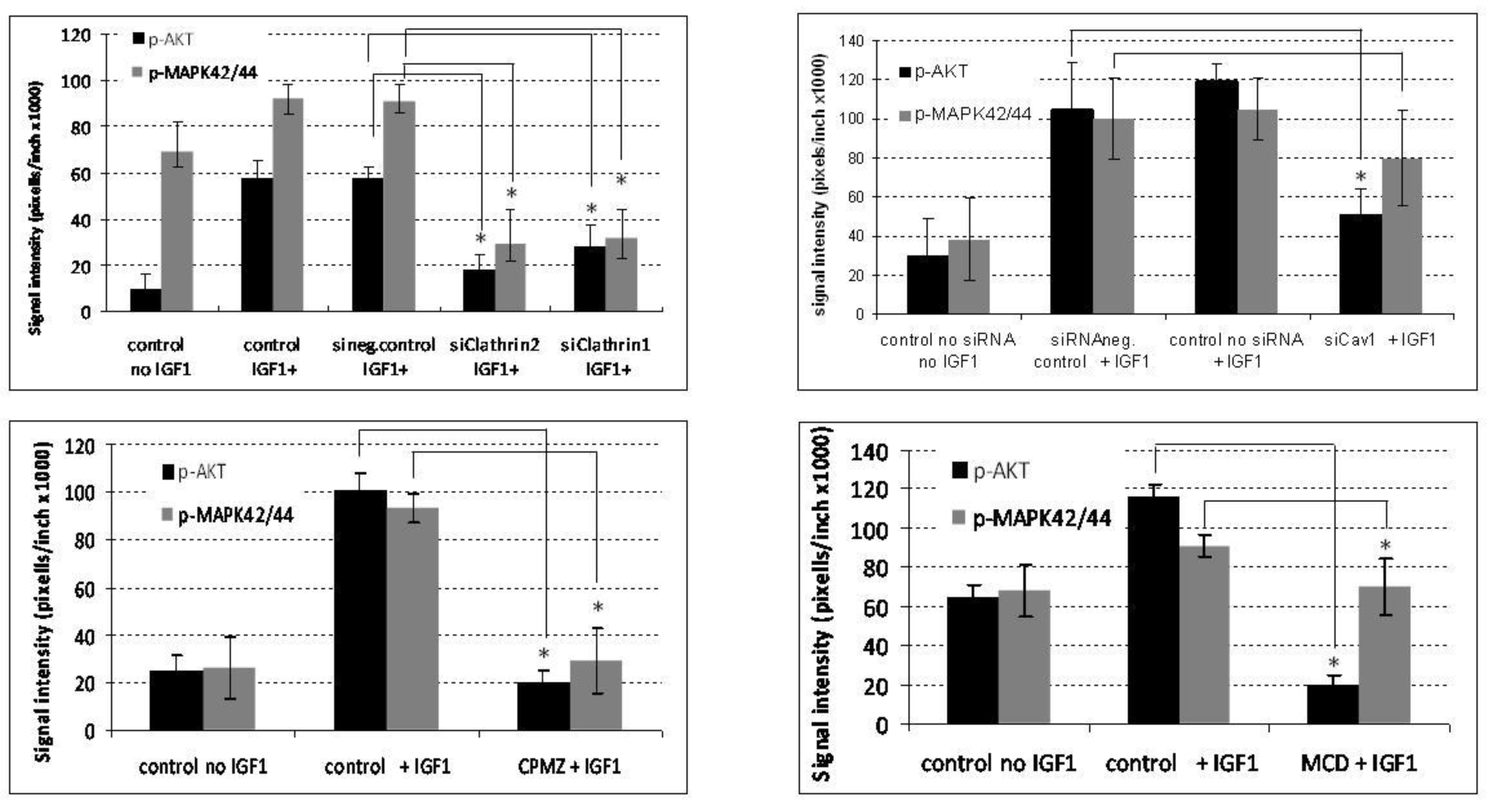

Supplement: Figure S2 — Densitometric analyses of WB shown in figure 2C and D of effects of CAV1 or clathrin inhibition/interference on the IGF1R signaling pathway. The inhibition of phosphorylation reached levels of over 60% reduction (p<0.05). The results obtained with the A673 and A4573 cell lines were identical to those described for TC71 (data not shown). Data presented is representative of 3–6 independent experiments. (TIF) [file pone.0019846.s002.tif]

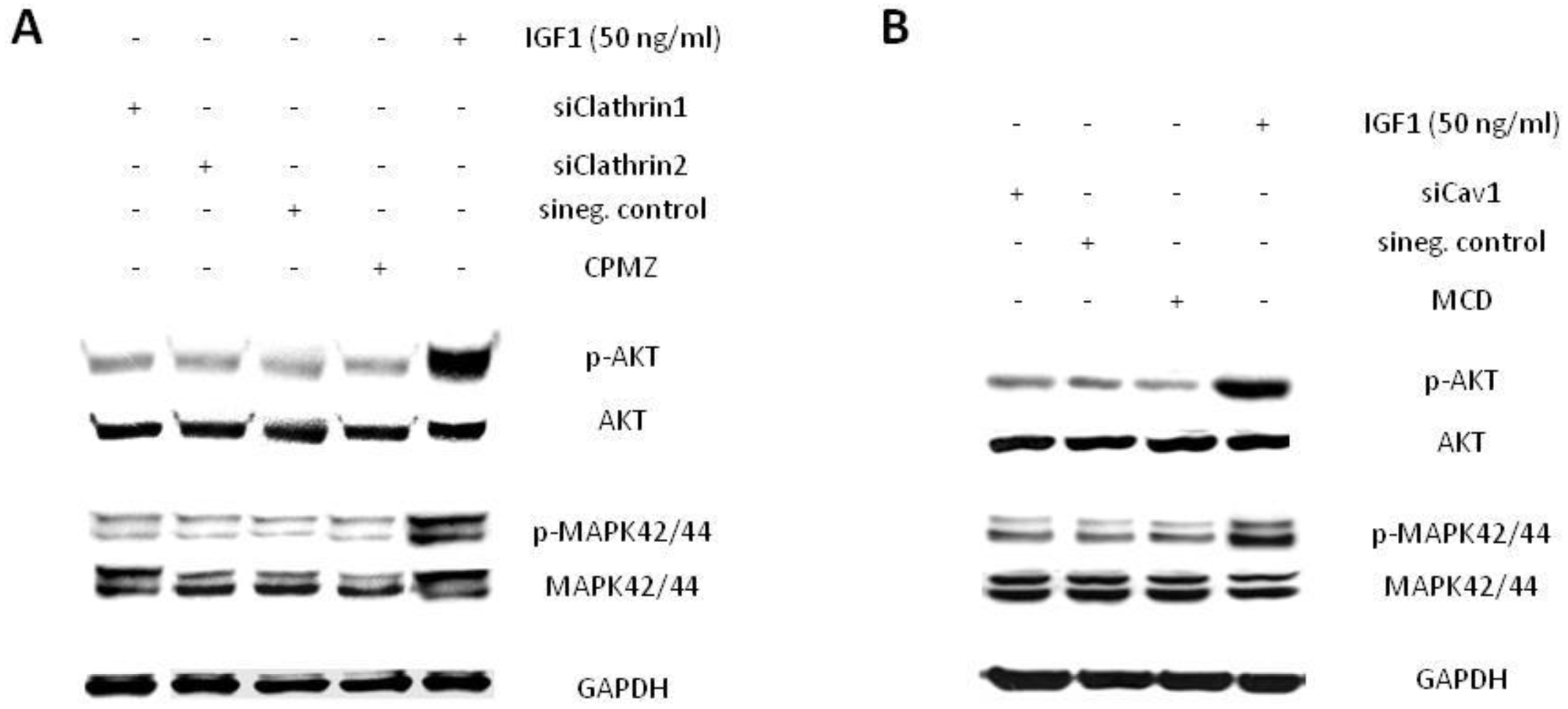

Supplement: Figure S3 — Effects of CAV1 or clathrin inhibition/interference on the IGF1R signaling pathway. Ewing's sarcoma TC71 cells were starved overnight, stimulated with IGF1 (50 ng/ml) for 15 minutes, and then proteins were extracted and WB was performed, as described in the Material and Methods section. A) Effects of clathrin inhibition/interference. B) Effects of Cav1 inhibition/interference. The phosphorylation of AKT and MAPK proteins in siRNA- or drug-treated cells without IGF1 treatment showed that siRNA or drug treatment alone do not interfere with protein phosphorylation. 3 independent experiments were performed and the final data presented selected as the most representative of the results obtained. (TIF) [file pone.0019846.s003.tif]

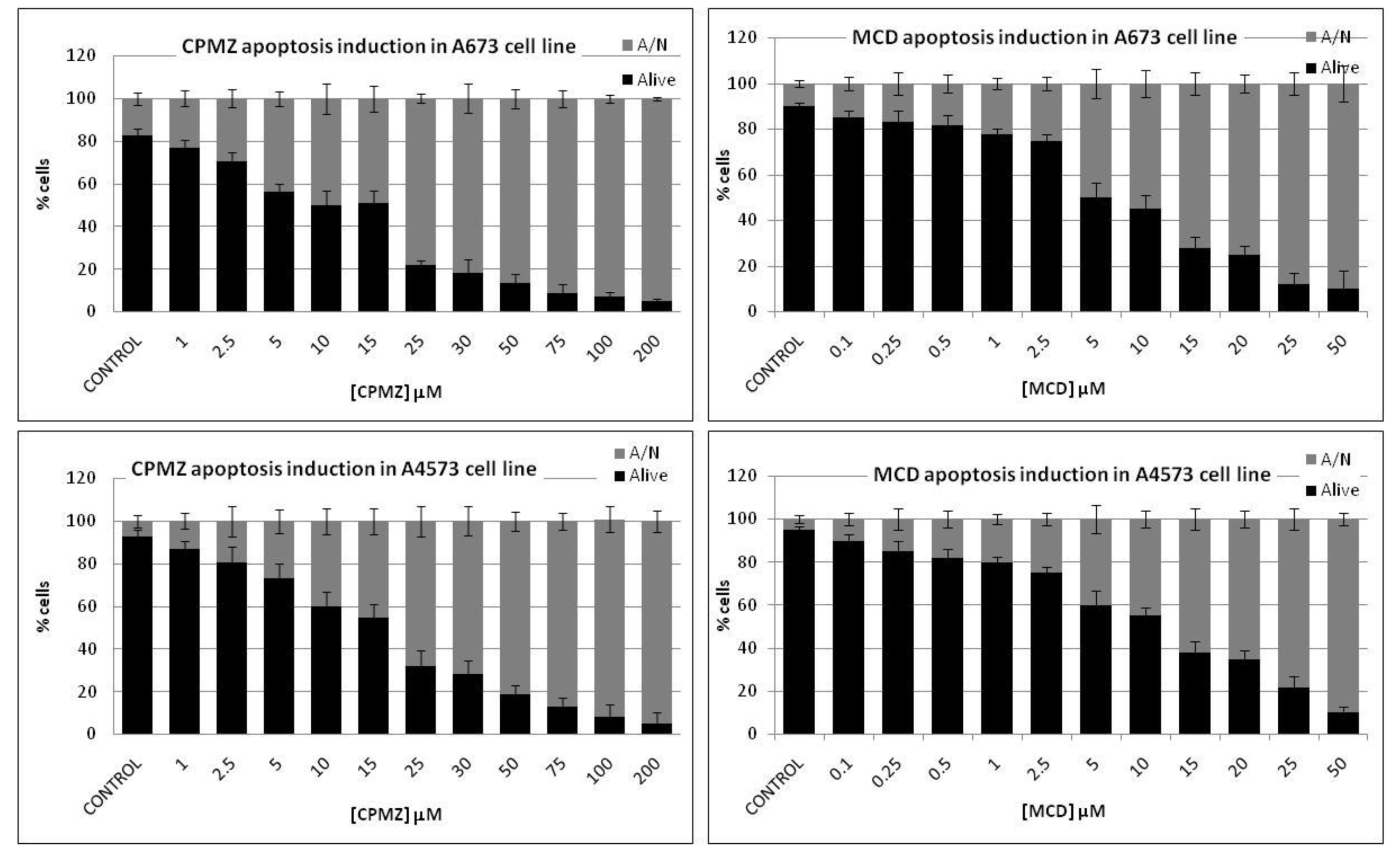

Supplement: Figure S4 — Effects of CAV1 or clathrin inhibition on apoptosis in the ES cell lines A673 and A4573. Ewing's sarcoma cells were grown until they reached 30–40% confluence, treated with MCD/CPMZ for 72 hours, and then apoptosis was measured by flow cytometry, as described in the Materials and Methods section. Drug treatment induced apoptosis in a dose-dependent manner in all ES cell lines, mainly inducing late apoptosis, with few cells undergoing necrosis. Data presented as the average of 4–6 independent replicates. (TIF) [file pone.0019846.s004.tif]

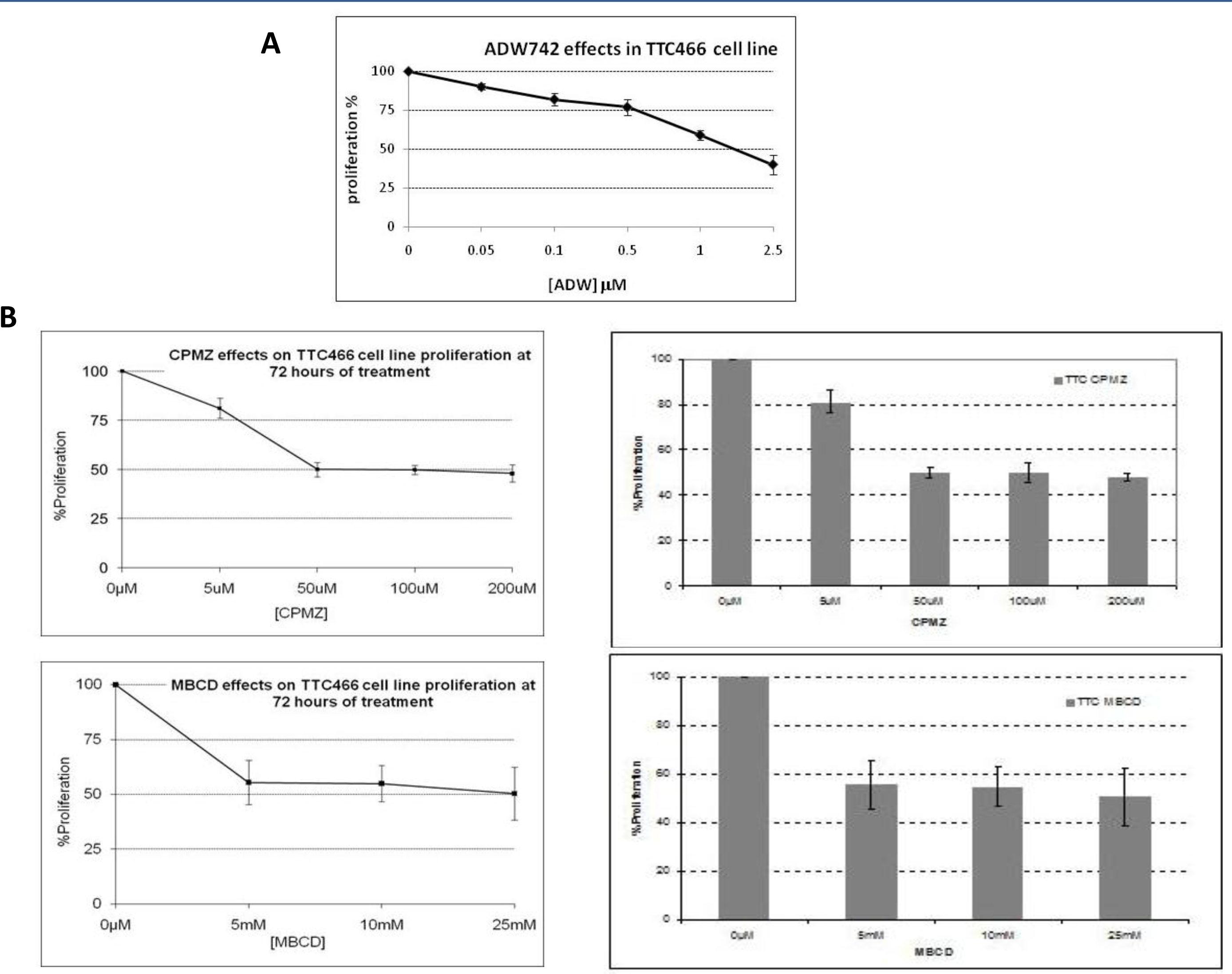

Supplement: Figure S5 — Effects of CAV1 or clathrin inhibition on an IGF-1-independent ES cell line. Ewing's sarcoma TTC466 cells were grown until they reached 30–40% confluence, treated with MCD/CPMZ for 72 hours, and then proliferation was measured with the MTT assay, as described in the Materials and Methods section. A) Characterization of the IGF1 proliferation-driven signaling dependence of the TTC466 cell line, as already published by Martins el al. Cancer Res. 2008; 68(15):6260–70. B) CPMZ or MCD treatment. CAV1 or clathrin inhibition hardly affected ES cell line proliferation. The IC50 of proliferation was much higher than with the 3 other cell lines studied (5–10x), showing that the toxicity of CPMZ and MCD is related to IGF1R endocytosis inhibition and not due to non-specific secondary effects of these drugs. Data presented as the average of 4-6 independent replicates. (TIF) [file pone.0019846.s005.tif]

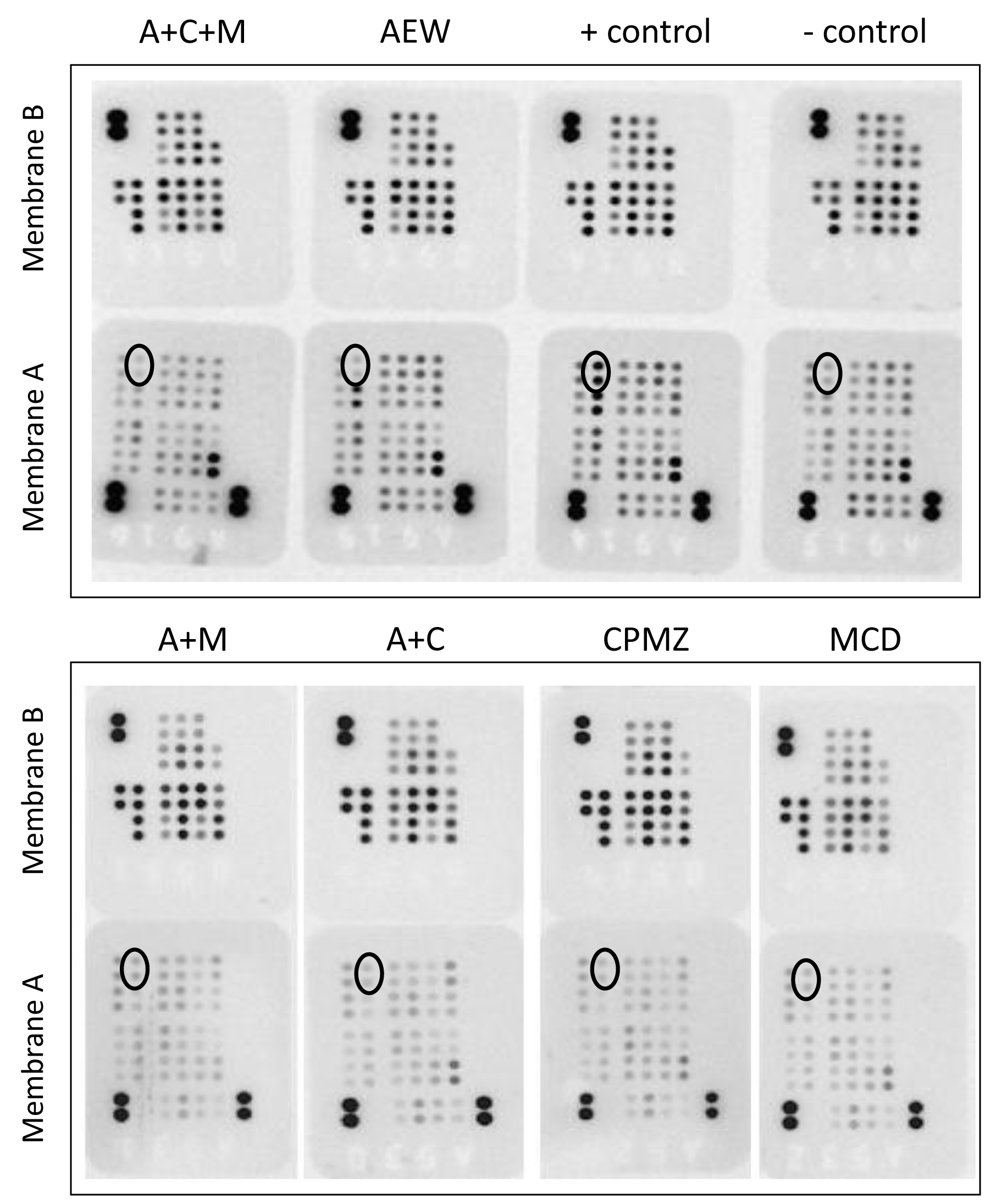

Supplement: Figure S6 — Effects of IGF1R tyrosine kinase inhibition combined with CAV1 and/or clathrin inhibition on the ES cell phospho-proteome. Images of the p-array membranes of all samples studied. A: AEW, C: CPMZ and M: MCD treatments; + control: IGF1 stimulation without drug treatment; - control: basal conditions, without IGF1 stimulation. Data presented is representative of 3 independent experiments. (TIF) [file pone.0019846.s006.tif]
